# Supplementary figures and images for: Wild-type IDH1 inhibition enhances chemotherapy response in melanoma
Source: J Exp Clin Cancer Res. 2022 Sep 24;41:283. doi: 10.1186/s13046-022-02489-w (PMC9509573; doi:10.1186/s13046-022-02489-w)

# Supplementary Figure-1.

**A**

A375

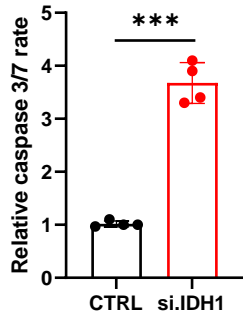

**B**

SK-MEL-28

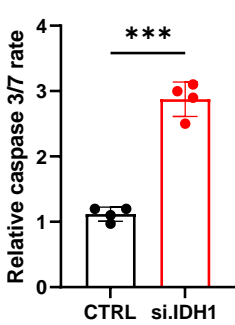

**C**

A375

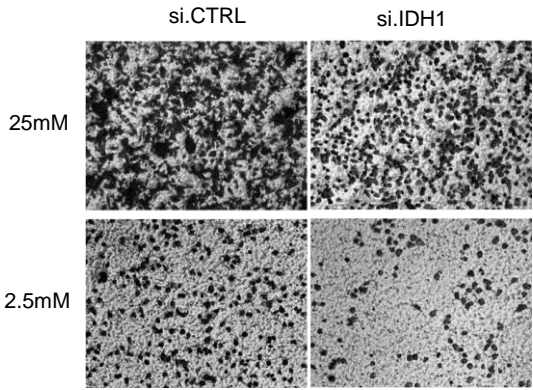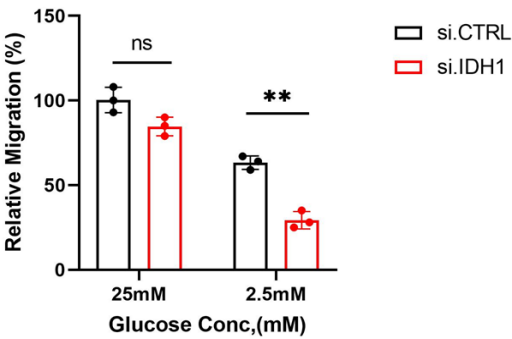

**D**

SK-MEL-28

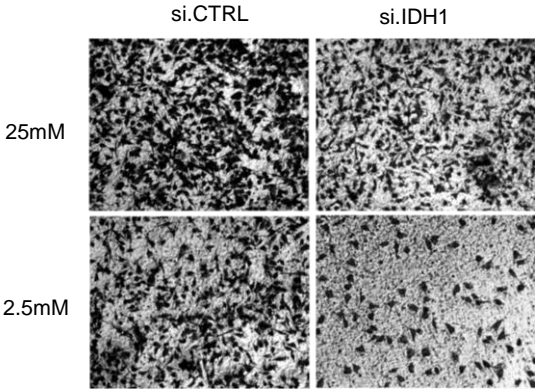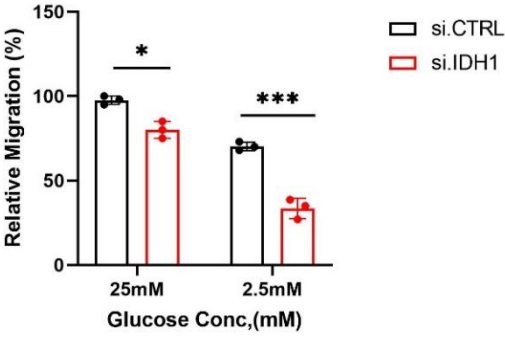

Supplement: Supplementary file 1 — Additional file 1: Supplementary Figure 1. IDH1 promotes cell migration under glucose limitation in melanoma cells. A] and B] Caspase3/7 activity was measured in A375 and SK-MEL-28 cells after 48 hours silencing IDH1 compared to control under low glucose concentration (2.5 mM). C] Representative cell images of A375 (4X magnification) and quantification of transwell migration under the indicated conditions after silencing IDH1 compared to control. D] SK-MEL-28 cell images (4X magnification) and quantitation of transwell migration under the indicated glucose concentrations. Each data point represents the mean ± SEM of at least three independent experiments. N.S., nonsignificant; *, P < 0.05; **, P < 0.01; ***, P < 0.001. [file 13046_2022_2489_MOESM1_ESM.pdf]

# Supplementary Figure-2.

A

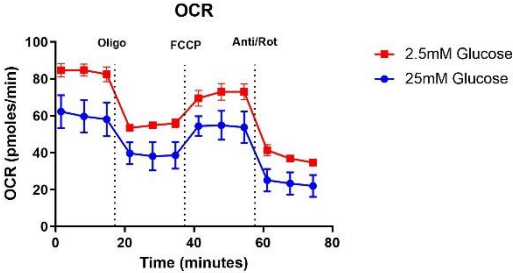

B

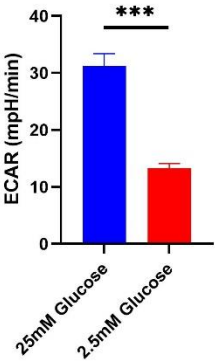

C

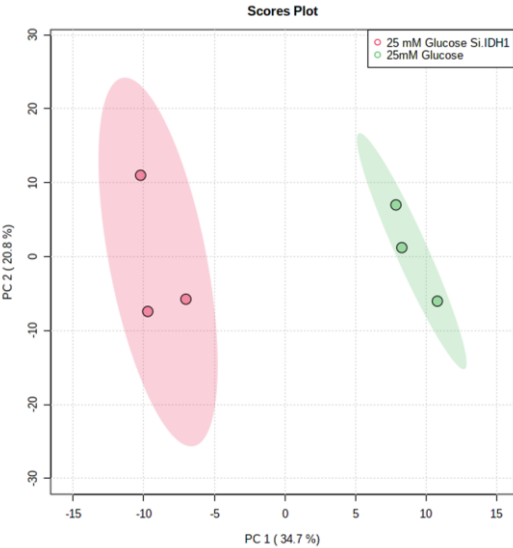

D

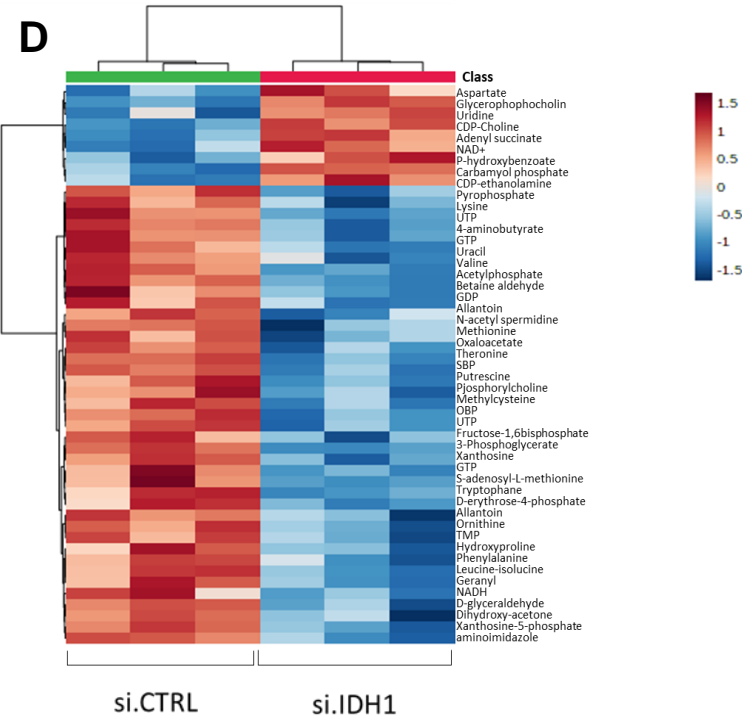

E

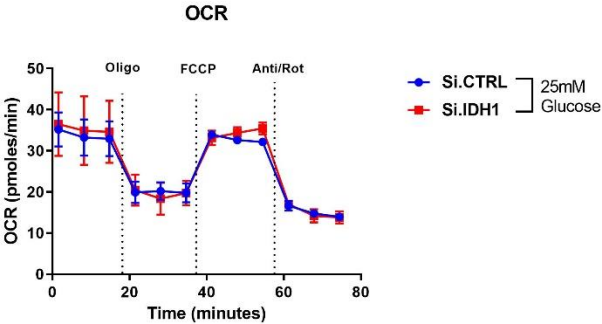

Supplement: Supplementary file 2 — Additional file 2: Supplementary Figure 2. IDH1 supports mitochondrial function under glucose limitation. A] Representative OCR tracing in A375 melanoma cells cultured under the indicated glucose concentrations for 24 hours and B] Extracellular acidification rate (ECAR) response of A375 cells under the indicated conditions. C] PCA of metabolites analyzed by LC-MS/MS performed on A375 cells under high (25 mM) and low (2.5 mM) glucose (n = 3 samples). D] A heatmap of the top 50 metabolites with the greatest changes in A375 cells (n = 3 independent samples) under 25 mM glucose. The scale is log 2 fold-change. E] Representative OCR tracing in A375 melanoma cells cultured under the indicated glucose concentrations for 24 hours. [file 13046_2022_2489_MOESM2_ESM.pdf]

# Supplementary Figure-3.

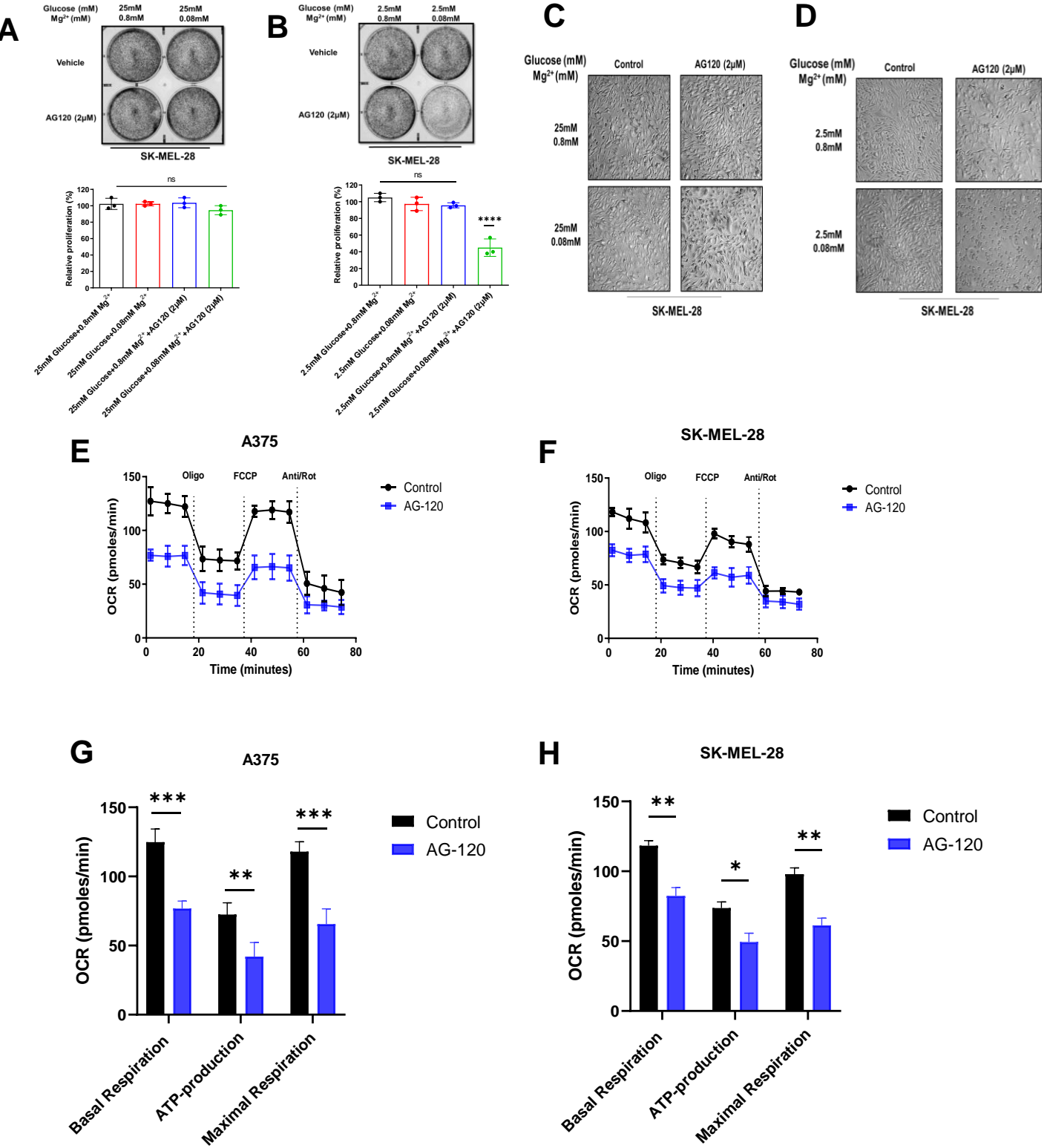

Supplement: Supplementary file 3 — Additional file 3: Supplementary Figure 3. AG-120 is a potential wtIDH1 inhibitor under glucose limitation in melanoma cells. A] Representative images of colony formation assays for cells cultured under high (25 mM) and B] low glucose (2.5 mM) in the SK-MEL-28 cell line. The cells were treated with vehicle control or AG-120 (2 μM) under the indicated conditions for 9 days. Quantitation (%) is shown in the graph at the bottom. C] Cells cultured under high glucose, or D] low glucose were captured by phase-contrast imaging (4X magnification), after treatment with vehicle control or AG-120 (2 μM) for 4 days. Representative oxygen consumption rate (OCR) in A375 E] and SK-MEL-28 F] cell lines cultured in 2.5 mM glucose and treated with vehicle or AG-120 for 36 hours. G] and H] Basal mitochondrial respiration, ATP production, and maximal mitochondrial respiration of the A375 and SK-MEL-28 cells treated with vehicle or AG-120 for 36 hours. Each data point represents the mean ± SEM of three independent experiments. N.S., nonsignificant; *, P < 0.05; **, P < 0.01; ***, P < 0.001. [file 13046_2022_2489_MOESM3_ESM.pdf]

# Supplemental Figure-4.

**A**

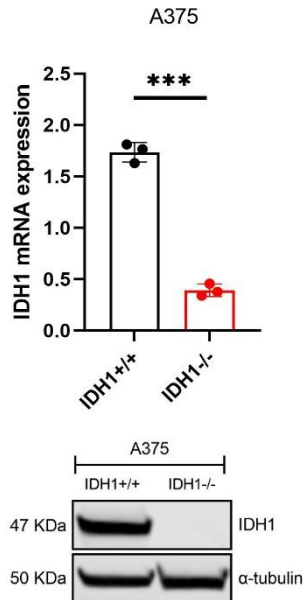

**B**

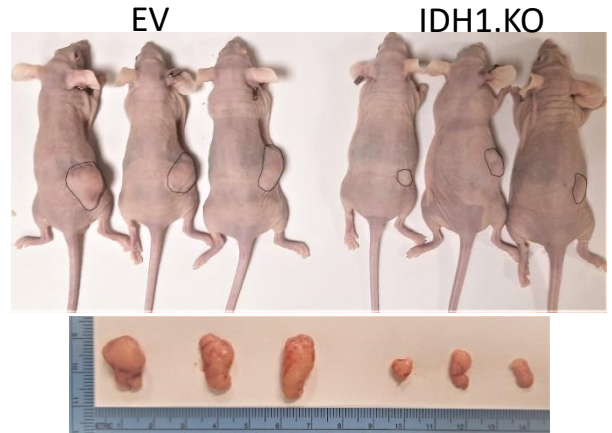

**C**

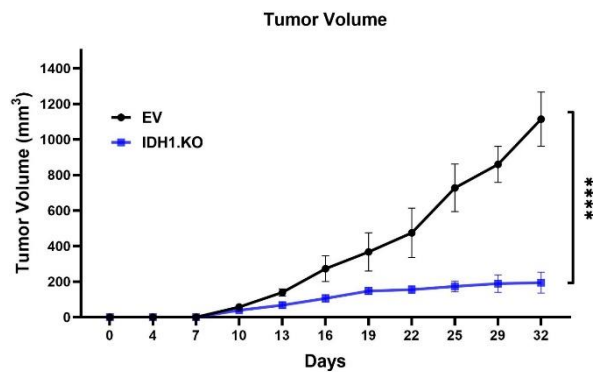

**D**

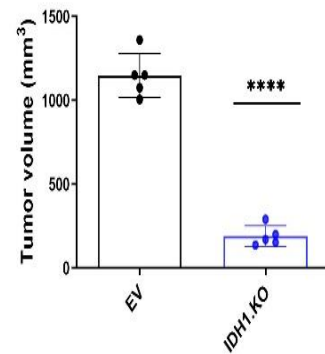

**E**

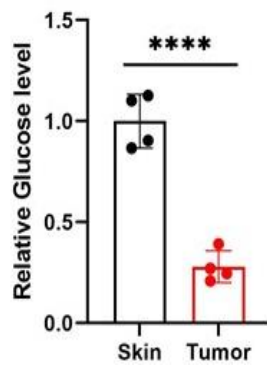

**F**

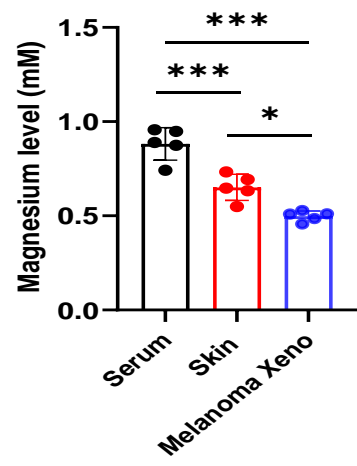

Supplement: Supplementary file 4 — Additional file 4: Supplementary Figure 4. IDH1 knockout suppresses tumor growth in vivo. A] Relative mRNA levels, normalized to mRNA levels of 18S; Western blot analysis of IDH1 expression after IDH1 knockout by CRISPR/Cas9 (IDH1.KO) compared to control (IDH1.EV) A375 cells. B] Mice were injected with IDH1.EV and IDH1.KO A375 cells (n = 5 per group) and tumor sizes were monitored for 5 weeks. Images of tumors at the end of the experiment are shown. C] Tumor volumes of IDH1.EV and IDH1.KO A375 xenografts. D] Histograms show tumor volumes with IDH1.EV and IDH1.KO A375 xenografts at the end of the experiment. E] Relative glucose levels in adjacent skin and xenograft. F] Relative free magnesium levels in skin, xenografts and serum. Each data point represents the mean ± SEM. *, P < 0.05; **, P < 0.01; ***, P < 0.001; ****, P < 0.0001. [file 13046_2022_2489_MOESM4_ESM.pdf]

# Supplementary Figure-5.

A

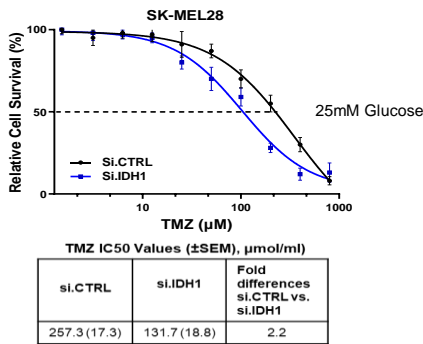

B

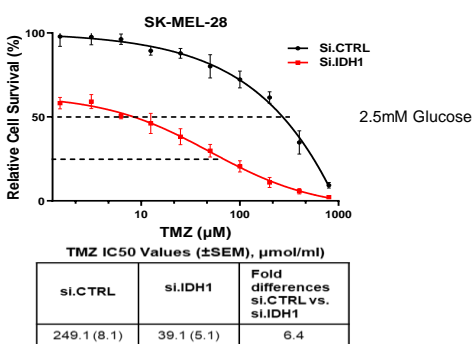

C

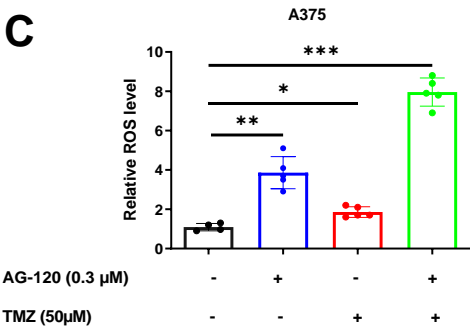

D

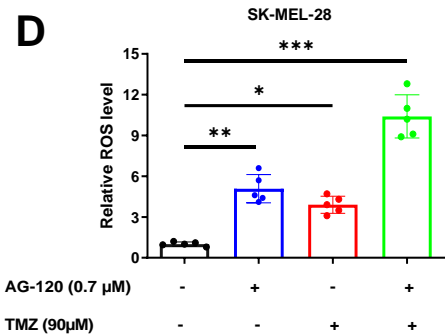

E

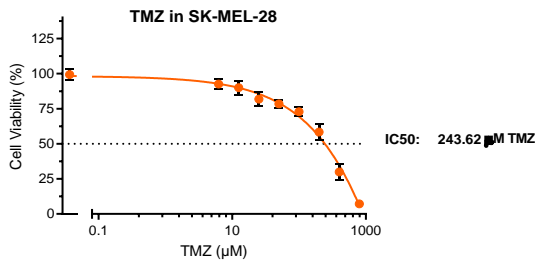

F

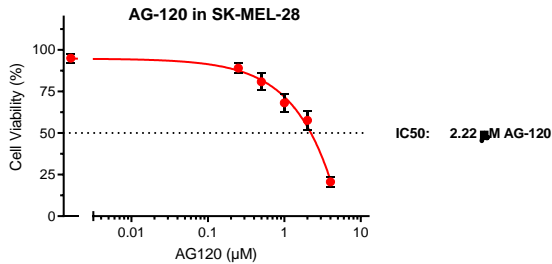

G

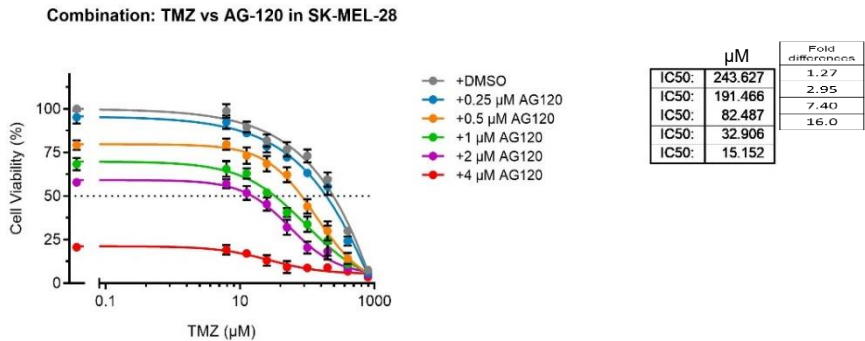

H

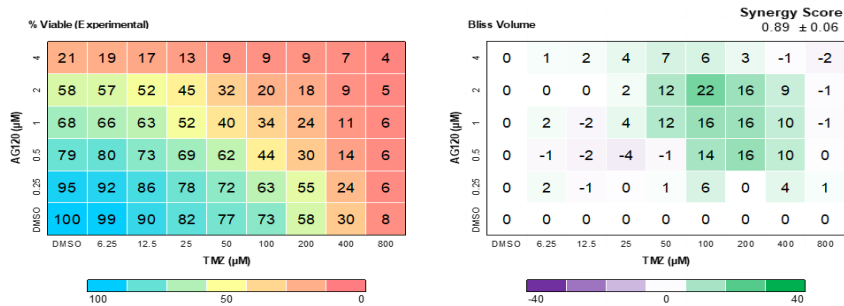

Supplement: Supplementary file 5 — Additional file 5: Supplementary Figure 5. In vitro response of melanoma cells to treatment with TMZ in combination with AG-120. A] Silencing IDH1 followed by treatment with TMZ for 5 days under high glucose (25 mM) and B] low glucose (2.5 mM) in SK-MEL-28 cells; IC50 values are provided. C] Relative ROS levels after 48 hours in A375 cell line under low glucose combined with TMZ and AG-120. D] Relative ROS levels after 48 hours in SK-MEL-28 cells under low glucose combined with TMZ and AG-120. E] Cell viability of SK-MEL-28 cells, treated with the indicated doses of TMZ; IC50 values are provided. F] Cell viability of SK-MEL-28 cells treated with indicated doses of AG-120 for 6 days. IC50 values are provided. G] Drug sensitivity in SK-MEL-28 cells under low glucose, with varying concentrations of TMZ and AG-120, cultured for 5 days under low glucose. IC50 results are provided. H] Drug matrix heatmap 5 × 8 (AG-120 and TMZ) grid showing percent viability and Bliss Independence scores in SK-MEL-28 cells cultured under 2.5 mM glucose for 5 days. Positive values reflecting synergism appear green on the heatmap (Bliss volume ≥ 10). All treatments with AG-120 were carried under low glucose (2.5 mM) and low Mg2+ (0.08 mM). [file 13046_2022_2489_MOESM5_ESM.pdf]

# Supplementary Figure-6.

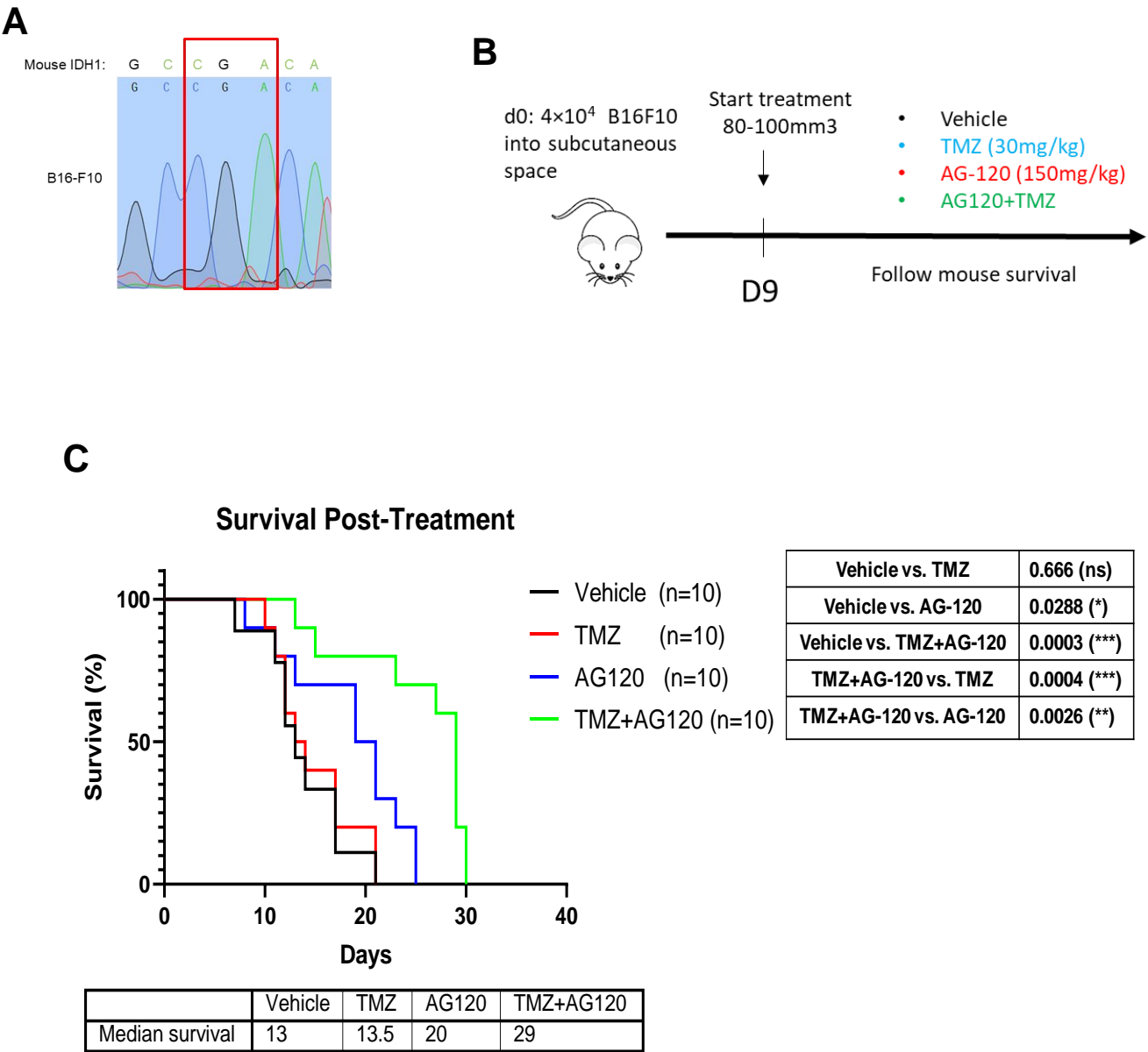

Supplement: Supplementary file 6 — Additional file 6: Supplementary Figure 6. Treatment of mice bearing B16-F10 tumors with TMZ in combination with AG-120. A] Sanger sequencing of PCR amplicons correlated with codon 132 of the IDH1 gene in B16-F10 murine melanoma cells. B] Schematic represents the treatment model after 4 × 10^4^ B16-F10 melanoma murine cells were injected subcutaneously into the flanks of C57BL/6 J recipient mice. After 9 days, when tumors reached 80-100 mm3, mice were divided into four groups and treated with i) Vehicle; ii) TMZ (30 mg/kg intraperitoneal once a day); iii) AG-120 (150 mg/kg orally twice a day); and iv) AG-120 + TMZ (150 mg/kg orally twice a day + 30 mg/kg intraperitoneal daily). C] Survival data of C57BL/6 J mice are represented by Kaplan-Meier curves. Significance between each group was determined using the log-rank test. [file 13046_2022_2489_MOESM6_ESM.pdf]
